# Insights into Anthropogenic Micro- and Nanoplastic Accumulation in Drinking Water Sources and Their Potential Effects on Human Health

**DOI:** 10.3390/polym15112425

**Published:** 2023-05-23

**Authors:** Maria Râpă, Raluca Nicoleta Darie-Niță, Ecaterina Matei, Andra-Mihaela Predescu, Andrei-Constantin Berbecaru, Cristian Predescu

**Affiliations:** 1 Faculty of Materials Science and Engineering, University Politehnica of Bucharest, 313 Splaiul Independentei, 060042 Bucharest, Romania; maria.rapa@upb.ro (M.R.); ecaterina.matei@upb.ro (E.M.); andra.predescu@upb.ro (A.-M.P.); cristian.predescu@upb.ro (C.P.); 2 Physical Chemistry of Polymers Department, Petru Poni Institute of Macromolecular Chemistry, 41A Grigore Ghica Voda Alley, 700487 Iasi, Romania

**Keywords:** drinking water treatment plant, microplastics, nanoplastics, tap water, bottled water, quantification, toxicological effect

## Abstract

Anthropogenic microplastics (MPs) and nanoplastics (NPs) are ubiquitous pollutants found in aquatic, food, soil and air environments. Recently, drinking water for human consumption has been considered a significant pathway for ingestion of such plastic pollutants. Most of the analytical methods developed for detection and identification of MPs have been established for particles with sizes > 10 µm, but new analytical approaches are required to identify NPs below 1 µm. This review aims to evaluate the most recent information on the release of MPs and NPs in water sources intended for human consumption, specifically tap water and commercial bottled water. The potential effects on human health of dermal exposure, inhalation, and ingestion of these particles were examined. Emerging technologies used to remove MPs and/or NPs from drinking water sources and their advantages and limitations were also assessed. The main findings showed that the MPs with sizes > 10 µm were completely removed from drinking water treatment plants (DWTPs). The smallest NP identified using pyrolysis–gas chromatography–mass spectrometry (Pyr-GC/MS) had a diameter of 58 nm. Contamination with MPs/NPs can occur during the distribution of tap water to consumers, as well as when opening and closing screw caps of bottled water or when using recycled plastic or glass bottles for drinking water. In conclusion, this comprehensive study emphasizes the importance of a unified approach to detect MPs and NPs in drinking water, as well as raising the awareness of regulators, policymakers and the public about the impact of these pollutants, which pose a human health risk.

## 1. Introduction

Global plastic production, including fossil-based plastics, post-consumer recycled plastic, and bio-based plastics rose to 390.7 million tons in 2021 [1]. From approximately 300 million tons of plastics manufactured annually [2], it was estimated that 13 million tons of plastic waste enter rivers and oceans [3]. Due to the resistance of plastic waste to degradation, it currently causes a serious pollution for the environment [4]. The management of plastic waste is very deficient, for example, 9% of global plastic waste is recycled, 12% incinerated, and 79% disposed in landfills, dumps, and oceans [5]. Only 5.5 million tons of post-consumer recycled plastics were reintroduced to the European economy in 2021 [1]. 

In the aquatic environment, due to the continuous abiotic degradation, plastic items (macroplastics) eventually break down into smaller fragments less than 5 mm in size to a few nanometers, typically known as microplastics (MPs) and nanoplastics (NPs) [3,6,7,8,9,10]. The recognized size ranges for MPs and NPs are still confusing. According to ISO/TR 21960:2020, “microplastic” stands for “any solid plastic particle insoluble in water with any dimension between 1 µm and 1000 µm (=1 mm),” and “nanoplastic” is defined as “plastic particles smaller than 1 µm” [11]. According to the Committee for Risk Assessment (RAC) and Committee for Socio-Economic Analysis (SEAC), “microplastic” means “particles containing solid polymer, to which additives or other substances may have been added, and where ≥1% *w*/*w* of particles have (i) all dimensions 0.1 µm ≤ x ≤ 5 mm, or (ii) a length of 0.3 µm ≤ x ≤ 15 mm and length to diameter ratio of >3” [12]. The term “particles” is “a minute piece of matter with defined physical boundaries; a defined physical boundary is an interface” [12]. Some authors defined the dimension of MPs according to the ISO definition [6,8], whilst others reported a classification between <5 mm and 1 µm [3,9,13]. Other authors categorized NPs as plastic particles with dimensions < 100 nm [6,7,10]. In this paper, we assumed that plastic particles with dimensions ranging between <5 mm and 1 µm are MPs, while those measuring below 1 µm are NPs. The “microplastic” term should not be used for natural polymers that have not undergone chemically modification, except in the case of hydrolysis [12]. In accordance with their origin, MPs could be classified as primary (resulting from anthropogenic activities, cosmetics, textiles, personal care products) and secondary (derived from fragmentation of primary ones) [14]. MPs possess a hydrophobic nature and a morphology of microbeads, fibers, foils, pellets or fragments, while NPs have colloidal behavior [15].

The presence of MPs and NPs has been documented in the aquatic environment [16,17,18,19,20,21,22], food [23,24,25,26], soil [27,28,29] and air media worldwide [30,31]. Artificial turf used in sports fields and the degradation of larger plastic pieces from commercial packaging waste are the primary contributors to the presence of MP pollution in European waters [32]. The increase number in the number of scientific publications on the occurrence, detection, characterization and impact of MPs and NPs on aquatic organisms and human health indicates the true importance of these pollutants [33]. It is expected that MPs and NPs may have unique reactivity and bioavailability for aquatic organisms [34]. MPs and NPs remain in the environment for a long time due to their chemical stability after entering water compartments and greatly affect the function of aqueous systems, being considered ubiquitous pollutants [35,36]. However, in a natural aquatic environment, the microorganisms immediately colonize the surface of MPs and NPs, which forms biofilm that alters not only the physicochemical characteristics of MPs/NPs but also their mobility, stability, bioreactivity, settlement, and fate in the environment [37,38,39]. The mechanism of biofilm formation on the surface of MPs in a water environment involves (i) the attachment of microorganisms to the surface of MPs, (ii) secretion of extracellular polymers (EPS) by microorganisms, and, (iii) multiplication of microorganisms [39]. This biofilm occurs on the surface of MPs and leads to their degradation through fragmentation.

Biodegradable polymers are plastic materials with high molecular weights that break down into H_2_O, CO_2_, and microbial biomass final products over time with the help of naturally occurring microorganisms [40]. It is important to note that there is a distinction between biodegradable plastics and bio-based plastics, which are obtained from renewable resources as an alternative to petroleum resources but may not be biodegradable [40]. Studies showed that if the conditions for assuring biodegradability are not met, the biodegradable plastics contribute to the generation of MPs and NPs like conventional plastic materials [41,42,43]. A promising approach for the total removal of MPs from aqueous media could be the use of microorganisms [44,45]. Unfortunately, 100% degradation of biodegradable materials cannot be reached under natural environments, so the occurrence of biodegradable MPs could be an additional threat to the environment [40,46,47]. According to Wei et al. [48], biodegradable polymers produce more MPs in aqueous environments compared to conventional polymers because they hydrolyze faster in basic environments.

Both conventional and biodegradable polymers can contain chemical contaminants such as UV filters [49], preservatives, per- and polyfluoroalkyl substances (PFAS) [50] or flame retardants [51,52,53], which are considered potential carriers of MPs due to their strong hydrophobicity [54]. The migration and deposition of chemical contaminants can occur due to continuous fragmentation and an abundance of aged MPs [55] increasing the potential risk to human health. In addition, MPs can bind or adsorb emerging pollutants from the water environment, such as pharmaceuticals and personal care products [13,56,57,58], heavy metals, polycyclic aromatic hydrocarbons [54], and antibiotics [59,60], based on the specific polymer type, chemical and physical properties of MPs. Compared to MPs, NPs are considered to pose an increasing potential risk to ecosystems and human populations due to their higher specific surface-area-to-volume ratio, thus increasing the potential source of chemical contaminants [61].

According to the World Health Organization (WHO), people should consume between 3.7 L and 2.7 L of liquids per day depending on their body weight [62]. Most of these liquids comes from tap water or drinks made with tap water. A research paper reported average concentrations of 94.37 MPs/L in bottled water and 4.23 MPs/L in tap water [63]. Recently, WHO has called for a more advanced assessment of plastic pollution in the environment, following the fact that small plastic particles have been identified in 90% of bottled water [64]. This includes the identification of the sources and pathways of MPs, as well as the development of effective strategies to reduce plastic pollution and mitigate its impact on human health. Human ingestion of MPs from water bottles can occur through the use of disposable water bottles, bottles made from recycled plastic, or glass bottles [26]. The US Food and Drug Administration (FDA) has proposed tolerable levels of ingestible contaminant from recycled plastic to be less than 1.5 µg/person/day [10]. However, the potential daily ingested dose imposed by the FDA was exceeded for children and adults, where intakes of 87.8 mg/kg/body weight and 40.1 mg/kg/body weight, respectively, were reported [65]. According to Cox et al. [63], children and adults consume an average of approximately 79,828 MPs and 97,827 MPs per year through drinking water. Other studies have reported an annual intake of 2550–5100 MPs [62] or even 4.1 × 10^4^ items [66] for the average consumer. The most commonly ingested types of MPs from drinking water were fibers and fragments [63].

The sources of drinking water for human consumption are tap water and bottled drinking water. Tap water is supplied by drinking water treatment plants (DWTPs), which play a vital role in ensuring water safety and meeting the social standards for human consumption [67,68]. Recently, the presence of MPs in DWTP was reported [69,70,71] and removal methods are still under investigation. From DWTPs, the unremoved MPs can enter water compartments used for daily human drinking water consumption, causing potential toxicological effects through ingestion, dermal exposure and inhalation [72,73,74,75]. Despite water being essential for life, few studies have been reported on the removal of MPs and NPs from drinking water sources [75] and their impact on human health. The risk of MPs uptake from drinking water is currently unpredictable. Furthermore, these plastic particles add to the plastic potentially ingested through the consumption of other foods/beverages, including sea salt, beer, food and seafood [76]. Until recently, significant knowledge regarding the detection and identification of these plastic pollutants, especially those in complex matrices present in raw and treated drinking water, was not available. This situation is a consequence of various factors, including the varied chemical composition and surface properties of MPs/NPs [77]. Additionally, distinguishing between NPs and natural matter can be challenging, and the aggregation of NPs can lead to changes in the solution ionic strength, which further complicates advanced characterization [78]. According to reports from the Agency for Toxic Substances and Disease Registry (ATSDR), polystyrene (PS) and polyvinyl chloride (PVC) are among the most carcinogenic contaminants [79]. The toxicological effects associated with MPs/NPs present in drinking water depend on the duration and intensity of exposure, as well as the susceptibility, gender and age of the host [73]. However, the toxicological mechanisms by which MPs/NPs affect human health are still unknown.

Nevertheless, the investigation of naturally occurring MPs and NPs in drinking water coming from treatment plants, tap and commercial water bottles in terms of quantification, toxicological effects on human health and methods of their elimination has not been fully explored been fully explored. In a recent review conducted for the period 2016–2021, it was reported that only nine studies had been devoted to the particle sizes and concentrations of MPs detected in bottled drinking water [73].

The main objectives of this review are: (a) to evaluate the main technologies applicable for removal of MPs/NPs from drinking water sources, such as DWTP, tap and bottled drinking water, (b) to review the innovative analytical methods used for the detection of MPs/NPs occurring in drinking water sources, and (c) to assess the potential toxicological risks associated with the human consumption of MPs/NPs from drinking water sources.

## 2. Methodology

Our search strategy involved the analysis of the most recent papers published in the last 10 years using the Web of Science database, the search terms being “microplastics/nanoplastics”, “microplastics/nanoplastics in drinking water”, “microplastics/nanoplastics in bottled water”, “toxicological effect of microplastics/nanoplastics”. The bibliographic survey was conducted by selecting papers based on their titles and abstracts, and later, analysis of the full-length articles.

**Figure 1:**
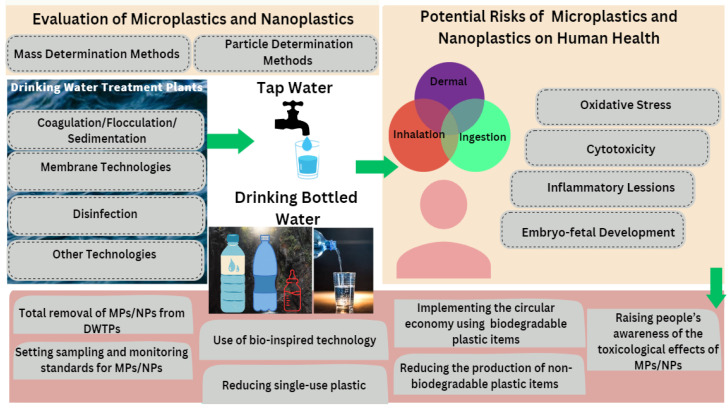
Overview of the review structure.

Figure 1 shows the overview of the review structure.

The first part of the review presents the conventional physicochemical methods used for removal of MPs and NPs from drinking water sources, such as coagulation–flocculation–sedimentation (CFS), filtration, ozonation, membrane filtration technology and adsorption, their performance and limitations, and new approaches for elimination of MPs and NPs.

Next, other physicochemical methods for removal of MPs and NPs, newly introduced or that may be suitably combined depending on the complexity and diversity of MPs/NPs in liquid media, are discussed. Multiple techniques are usually required to obtain information about the concentration, chemical identification, and shape of NPs. In this section, the newest methods based on mass determination and particle measurement are discussed with respect to the concentration, morphology and polymer type of MPs/NPs detected in DWTP, tap water or bottled drinking water. Methods based on mass determination are pyrolysis–gas chromatography–mass spectrometry (Pyr-GC/MS) and thermal desorption–proton transfer reaction–mass spectrometry (TD-PTR/MS). General methods based on particle measurement include micro-Fourier-transform infrared (µ-FTIR) spectroscopy, micro-Raman (µ-Raman) spectroscopy, single particle inductively coupled plasma mass spectrometry (SP-ICP-MS), dynamic light scattering (DLS), scanning electron microscopy–energy dispersive X-ray spectroscopy (SEM-EDX), and transmission electron microscopy (TEM).

Subsequently, the potential toxicological effects of MPs/NPs from drinking water sources on human health are assessed based on the size and concentration of MPs/NPs, as well as other additives incorporated into bottled drinking water. Most studies have been conducted on the exposure of marine organisms to commercial PS NPs purchased from authorized institutions or polyethylene terephthalate (PET) NPs. This is because PS NPs have been detected in the marine environment [80] and PET is the most widely used material for manufacturing bottles and packaging [81,82]. Few studies have been dedicated to investigating the effects of NPs released into drinking water sources on human health.

Finally, this paper highlights the main legal, technical, and social measures necessary to reduce the risks associated with contamination of drinking water sources with MPs/NPs and summarizes the key conclusions.

## 3. Results and Discussions

### 3.1. Physicochemical Methods for Removal of Micro- and Nanoplastics from Drinking Water Sources

DWTPs are processes with multiple stages that provide safe drinking water for human consumption [83]. Conventional methods used to remove MPs/NPs from DWTPs include coagulation–flocculation–sedimentation (CFS), filtration [3,17,18,84], ozonation [19,85], and chlorination [85]. The treatment process in a DWTP influences the quality of the drinking water produced [72].

In this section, the most frequently used physicochemical methods for removing of MPs/NPs from drinking water sources have been assessed (Table S1). The effectiveness of MPs/NP removal from drinking water sources depends on various factors, such as their physicochemical parameters (concentration, molecular weight, size, shape, age), the adopted treatment method, the type and dosage of coagulant, the type of filtration, water source, as well as the presence of mixture of natural compounds, named natural organic matter (NOM) [72]. Also, the old and worn components from the DWTP could potentially be a potential source of MPs in drinking water [69]. Studies have reported the successful removal of intentionally added MPs/NPs in water [3,17,54,78,85], as well as those present in DWTPs [69,70,71]. Recently, it was demonstrated that MPs with sizes > 10 µm can be completely removed from DWTPs, and over 80% of the removed MPs had a dimension > 1 µm [86].

#### 3.1.1. Coagulation–Flocculation–Sedimentation (CFS)

Coagulation is an important conventional method used for the removal of pollutants from drinking water sources [54]. Most studies carried out for plastic contaminant removal from drinking water have used spherical engineered particles, especially PS (Table S1), without surface roughness or biofilm. A few studies have dealt with the monitoring of small MPs (<10 μm) in drinking water [3,17,78]. The presence of NPs in drinking water treatments has been reported [3], but their complete removal has not yet been revealed. The mechanism of PS NP removal was attributed to physical retention and straining processes [3].

PE MPs of three sizes (10–20 μm, 45–53 μm, and 106–125 μm), PS NPs (180 nm in size), and PS MPs (1.2 μm in size) were removed from drinking water by conventional treatment using coagulation–flocculation combined with sedimentation (CFS) and granular filtration in the presence of Al_2_(SO_4_)_3_ and diallyldimethylammonium chloride (polyDADMAC) as coagulant aid [17]. The results showed that granular filtration was more efficient than the CFS method, resulting in a 99.9% removal rate for polyethylene (PE) MPs with dimensions ranging from 106 to 125 μm [17]. Additionally, the presence of biofilm increased the efficiency of the CFS treatment, improving the removal of MPs from <2.0% to 16.5% [17]. The occurrence of biofilms can significantly modify MP characteristics (size, shape, and density) and subsequently the efficiency of water treatment. Other authors demonstrated that the biofilm formed on the surface of MPs/NPs increased the removal efficiency in column experiments conducted with aged sand from 43% to 77% [17,78]. The explanation consists in the presence of humic substances from NOM [87] facilitating the positively charged coagulants to adsorb NPs with a negative surface charge [88,89].

Regarding the concentration of coagulant, the data showed that 20 ppm is the typical maximum coagulant concentration used for effective drinking water treatment [17]. Aluminum sulfate [Al_2_(SO_4_)_3_] or alum, polyaluminum chloride (PACl) [(Al(OH)m Cl(3-m))n], polyDADMAC, aluminum chloride (AlCl_3_), and iron chloride (FeCl_3_) are the most commonly employed coagulants in water treatment [17,54,71,88]. The effect of two conventional coagulants, PACl and FeCl_3_, on NP removal from three bottled mineral waters and Lake Geneva was investigated [88]. It was found that at lower doses required for the coagulation of NPs, PACl was more effective than FeCl_3_ [88]. PACl is a conventional inorganic coagulant preferred for the treatment of water because it assures a low concentration of residual metal in treated water [83]. The efficiency of PACl was also reported by other authors [3].

The removal efficiency of engineered PE MPs from deionized water containing humic acid in the presence of FeCl_3_·6H_2_O and AlCl_3_·6H_2_O and sodium bicarbonate (NaHCO_3_) as a buffer followed by the ultrafiltration membrane technology was investigated by Ma et al. [54]. AlCl_3_·6H_2_O was found to be more effective in removing PE MPs than Fe-based salts. Both coagulant agents led to increased removal efficiency with decreased particle sizes of PE MPs. However, a lower removal efficiency of 25.83% ± 2.91% after coagulation and slight membrane fouling were recorded.

Other coagulants and coagulant aisd typically used in the CFS method, combined with filtration, are alum at a concentration of 20 ppm and polyDADMAC at 0.5 ppm [17]. PolyDADMAC is a type of high-molecular-weight polymer coagulant aid that helps in bridging and binding of particles, improving floc strength and achieving optimal floc size, leading to a higher rate of sedimentation. The authors reported a significant difference in the removal efficiency of the polyDADMAC coagulation aid (13.6% ± 6.8% for particle sizes 45–53 μm) compared to raw water and CFS treatment [17]. This behavior was explained by the ability of polyDADMAC to bind the particles, strengthen the floc, and increase the sedimentation rate [17].

Another strategy for removing engineered PE MPs with sizes ranging from 10 to 100 µm from synthetic water was to stain them with Nile red dye and use alum and alum combined with cationic polyamine-coated (PC) sand as coagulants [90]. PC sand (500 mg/L) combined with an alum dose of 20 mg/L showed the highest removal rate (92.7%) compared to using alum alone. The dimension, shape and surface morphology of the MPs played a significant role in the coagulation and flocculation mechanism. The order of MP removal was observed to be elongated-rough (ER) > elongated-smooth (ES) > spherical-rough (SR) > spherical-smooth (SS) based on the results of a flocculation kinetic study [90].

An overall efficiency of 82.1–88.6% was achieved in a DWTP that utilized various processes, such as coagulation–flocculation–sedimentation, sand filtration, ozonation, and granular activated carbon (GAC) filtration, for the removal of MPs [68].

Instead, the use of aluminum salt coagulant combined with sand filtration led to a removal efficiency of 93% ± 5% for MP removal (especially PS and polyester) in a DWTP from Spain [69]. In another study [70], the pre-disinfection with liquid chlorine gas, followed by the addition of alum coagulant (at a flow rate of 3000 L/h), purification with a pulse clarification system, sand filtration, and post-disinfection steps led to 85% removal efficiency in DWTPs.

Velasco et al. [71] compared the effectiveness of coagulant, sand filtration, and activated carbon (AC) for removing MPs such as synthetic fibers (cotton, viscose, and cellulose) from a DWTP. The results indicated that the use of PACl coagulant and AC filter led to a higher removal efficiency of MPs (97 ± 3%). This removal efficiency was compared to a lower rate of 89% when the coagulant was not used. Sand filtration already demonstrated high removal efficiency (95% without coagulant and 92% with coagulant), emphasizing the significance of this step [71]. However, other authors [68] reported that the sand filtration played no significant role in the removal of MPs. They achieved a removal efficiency of only 29.0–44.4% compared to coagulation/sedimentation methods. In terms of the shapes of MPs eliminated, it was found that fibers accounted for 96% when the coagulant, sand, and AC filtration were used [71]. In the case of CFS followed by ozonation integrated with GAC, the percentage of fiber elimination ranged from 51.6% to 78.9% [68].

Disadvantages associated with the coagulation treatments include reduced removal of MPs/NPs, high coagulant consumption [67], and increased presence of residual Al-based salts in the case of Al coagulants, which can have adverse effects on human health [91]. While the CFS technique alone is insufficient for removing plastic pollutants from drinking water, combining it with granular filtration techniques has been successful in removing MPs/NPs larger than 100 μm from DWTPs [17]. Special attention should be given to the configuration of DWTPs and operating parameters for the removal of both MPs and NPs from drinking water.

#### 3.1.2. Disinfection Technologies

During the process of disinfecting drinking water, MPs/NPs may pass through the filter and enter the municipal water supply network, eventually reaching consumers’ taps. To address this issue, ozonation and chlorination, which are frequently employed in water treatment plants, have been proposed as potential methods for degrading MPs/NPs [85].

**Figure 2:**
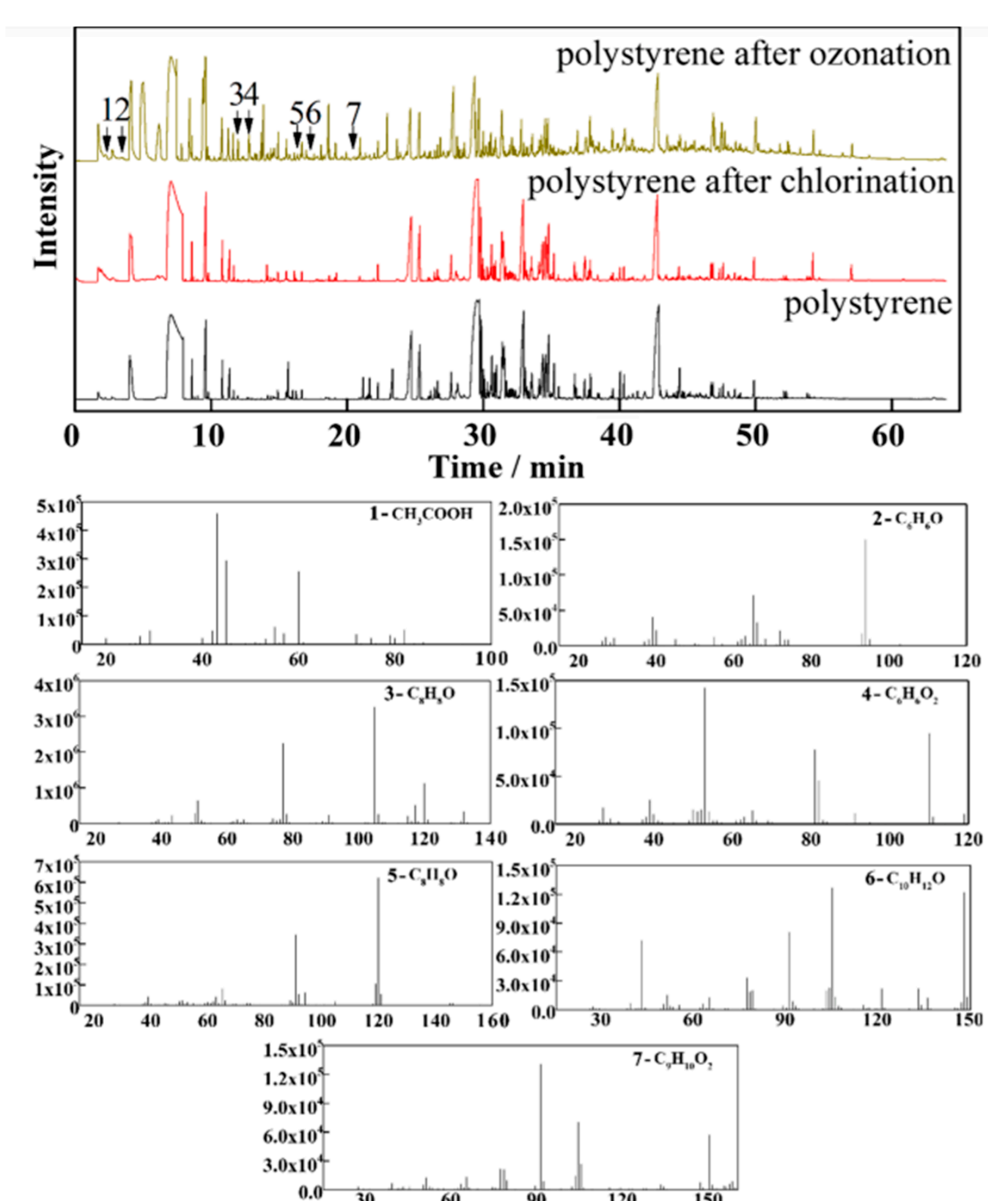
Pyr-GC/MS spectra of PS NPs, PS NPs after ozonation, PS NPs after chlorination, and the seven mass spectra from PS NPs after ozonation. Reprinted with permission from [85]. 1—acetic acid (CH_3_COOH), 2—phenol (C_6_H_6_O), 3—acetophenone (C_8_H_8_O), 4—hydroquinone (C_6_H_6_O_2_), 5—methylbenzaldehyde (C_8_H_8_O), 6—dimethyl acetophenone (C_10_H_12_O) and, 7—phenylpropionic acid (C_9_H_10_O_2_).

In one study [85], a concentration of 2.5 μg/L PS NPs in water was investigated to determine the effectiveness of ozonation and chlorination technologies for the degradation and mineralization of PS NPs. The average ozone dosage in drinking water was maintained at 4.1 mg/L, while 2.5 mg/L was the concentration of chlorine (in the form of hypochlorite salts) for chlorination. After 30 min, ozonation resulted in the removal of 96.3% of PS NPs, while chlorination only removed 4.2%. This significant difference was attributed to the increased hydrophilicity of PS NPs, which occurred due to the introduction of oxygen-containing groups on the surface during the ozonation treatment. It has been demonstrated through Pyr-GC/MS that ozonation is more effective than chlorination for the destruction of PS NPs from DWTPs. Chlorination, on the other hand, resulted in the formation of a shorter macromolecular chain due to a destruction of a small number of C-C bonds [85]. The Pyr-GC/MS spectrum of PS NPs after ozonation showed several new signals at retention times of 2.286 min, 3.393 min, 11.992 min, 12.091 min, 16.294 min, 17.137 min and 20.046 min, which corresponded to acetic acid (CH_3_COOH), phenol (C_6_H_6_O), acetophenone (C_8_H_8_O), hydroquinone (C_6_H_6_O_2_), methylbenzaldehyde (C_8_H_8_O), dimethyl acetophenone (C_10_H_12_O), and phenylpropionic acid (C_9_H_10_O_2_), respectively. In contrast, the spectrum of PS NPs after chlorination was identical to that observed before this treatment (Figure 2) [85].

In another paper, ozonation and three successive filtration media involving rapid sand, AC, and slow sand filtration were used for the removal of palladium (Pd)-labeled NPs from real DWTPs [78]. A minor impact related to transport through columns was observed when the NPs were pretreated with ozone. Slow sand filtration promotes biofilm formation and results in high efficiency for NP removal (99.5%).

Conversely, the amount of MPs slightly increased in the treated water after the ozonation process [68]. The authors explained the rise of MPs particles during ozonation as a result of the destruction of residual organic matter attached to the MPs, as well as the breaking of MPs due to the cutting force of the water flow.

#### 3.1.3. Adsorption

Adsorption is widely used for the removal of contaminants from aqueous solutions, being cost-effective and environmentally friendly [92,93,94]. The possible factors affecting the removal efficiency of MPs/NPs by adsorption include: pH, temperature, adsorbent types, dissolved organic matter (DOM), and ions [72]. pH and temperature are considered the two most important factors. pH affects the adsorption efficiency mainly by influencing the charge on the surface of MPs and the adsorbent, whereas temperature can influence the adsorbate diffusion rate and equilibrium capacity; higher temperatures led to high MPs adsorption [72].

GAC is the most widely used adsorbent material in the drinking water purification process due to its high surface area and porous structure [3,95]. The efficiency of positively charged PS NPs hydrazine was evaluated following sand and GAC filtrations in the main DWTP from Geneva (Switzerland), with and without coagulant agent [3]. An increased removal of PS NPs was found in the case of GAC filtration compared to sand filtration, explained by the adsorption capacity of GAG. By using PACl coagulant, the filtration efficiency increased up to 99.4% ± 1.1%, because the retention of large NPs aggregates was improved and surface charge of PS NPs was reduced, leading to repulsive forces between NPs and filter media, thus improving both their retention and removal.

Another study on the removal of NPs from DWTP by using CFS and sand/GAC filtration revealed a difference in the NP removal mechanism between those two techniques, NPs of 200 nm being more efficiently removed by CFS, while some smaller NPs (50 nm) were better removed by GAC filtration [84].

During the simulation of the dynamic adsorption process of engineered PS NPs by GAC filtration of drinking water, characterized by a surface area of 759 m^2^/g and pore volume of 0.357 cm^3^/g, a very stable structure of GAC was revealed, its abundant pore structure of 3.09 nm and good regeneration (90% after the first cycle) making this adsorbent effective for adsorption of PS NPs [14]. The addition of Ca^2+^ increased the ionic strength by reducing the electrostatic repulsion between PS NPs. This behavior favors the aggregation and retention of PS NPs in the GAC pores.

Recently, Sajid et al. [96] highlighted the role of metal–organic frameworks, bio-based nanomaterials, carbon-based nanomaterials, and layered double hydroxides as adsorbents for the elimination of MPs from aqueous media. In this vein, Martin et al. [97] exploited the ability of iron oxide nanoparticles (IONPs), synthesized via green chemistry methods and further coated with different hydrophobic or amphiphilic coatings, to separate, concentrate, and remove NPs from water by magnetic separation. Thus, 1000-multifilament yarn of 30 µm diameter, PE nurdles and PE fibers were used as models for the removal of NPs from freshwater and placed in contact with coated iron oxide nanoparticles (IONPs). All PE nurdles and fibers were collected with a simple 2-inch permanent NdFeB magnet [42].

However, at the end of their use, the adsorbents must be regenerated by removing the adsorbed MPs/NPs; otherwise, they present a potential risk of returning to the environment [72].

#### 3.1.4. Membrane Filtration Process

Membrane filtration is currently employed to remove emerging pollutants from contaminated waters [98]. For instance, vacuum-assisted filtration with an inorganic filter membrane (for example, Whatman Anodisc, 47 mm diameter, 0.2 µm pore size) was used to separate NPs from bottled and tap water [62]. Consequently, the total amounts of MPs particles found in tap water and plastic bottled drinking water ranged from 0.99 to 26 MPs/L [62]. In the case of the water deposited in glass bottles, the detected MPs were below the limit of quantification (LOQ) by µ-FTIR analysis. The composition of the detected MPs was: 38% PE, 25% PS, 22% and polypropylene (PP), polyamide (PA), and polyurethane (PU) in small amounts [62]. The most commonly used polymers for production of water bottles are PET for the body [16,99] and high-density polyethylene (HDPE) for the caps [100,101]. However, the source of MPs/NPs contamination depends on the bottle manufacturing technology.

Recently, Cherian et al. [102] reported the performance of three point-of-use (POU) water treatment devices containing GAC, ion exchange (IX), and microfiltration (MF) components used by consumers for optimal MP removal from drinking water. PE and PET as fragments and nylon as fibers were used as spiked MPs. The study showed that the POU device containing GAC and IX did not effectively remove MPs. The POU device containing GAC, IX and filtration membrane with pore size > 1 µm exhibited 78–86% and 94–100% removal for polyvinyl chloride (PVC) and PET, respectively, while, the best performance was reported in the case of the POU device including MF with pore size > 0.2 µm, GAC and IX, when 100% PVC and 94% PET were removed. High nylon fiber removal was observed in all POU devices. This study highlighted the importance of membrane- and small pore-associated membrane filtration process for MP removal.

Ultrafiltration units are often installed as a single filtration stage in newly constructed plants [103]. With pore sizes of about 10 nm, high NP removal efficiencies are expected through such treatment processes. Barbier et al. [104] showed, for the first time, that the nanofiltration process is more efficient than conventional methods (coagulation–flocculation, settling, sand filtration, ozonation, GAC filtration, UV treatment, and chlorination) used to remove MPs from DWTPs. Thus, by applying nanofiltration for MP removal from three DWTPs, the authors reported concentrations ranging from 7.4 to 45.0 MPs/L in the inlet water, while these decreased to 0.260 MPs/L in the case of outlet drinking water. The overall removal rate was >99%, and the MPs identified were PP, PE and PET.

Even though drinking water treatment processes should minimize the chances of MPs entering tap water, thus reducing their risk to consumers, membrane filtration can increase the number of MPs/NPs in some cases. MPs/NPs can be released in the membrane filtration step and further adsorb halogenated by-products resulting from chemical purification, thus becoming a secondary pollution during long-distance water transport [105]. For example, a concentration of MPs/NPs of 67.81 ng/L was found after using membrane filtration compared to 13.23 ng/L detected in tap water [106]. Membrane filtration is considered to exhibit a high potential ecological risk due to the membrane destruction during water filtration and clogged pores [106].

#### 3.1.5. Other Technologies

Another suitable approach for removing low-density polyethylene (LDPE) suspended on the water surface could be dissolved air flotation (DAF) water treatment. The purpose of DAF is to diffuse the air in the form of fine bubbles in the drinking water, followed by flotation of the suspended particles and their final removal by skimming [17]. The removal efficiency of MPs and NPs was evidenced by the analysis of sediment captured by filtration of 250–500 mL of raw water using filter membranes with various pore sizes (pore size of 25 nm for NPs with 180 nm particle size, and pore size of 200 nm for NPs with 1.2 μm, 10–20 μm, 45–53 μm, and 106–125 μm particle sizes) [17]. The filtration removal efficiency was not dependent on the size of the plastic particles. For example, by using five different sizes of fluorescent plastic particles, namely, 106–125 μm, 45–53 μm, 10–20 μm, 1.2 μm, and 180 nm, the corresponding removal efficiencies were reported to be 99.9% ± 0.1%, 97.0% ± 3.0%, 86.9% ± 4.9%, 94.9% ± 0. 4%, and 98.9% ± 0.7%, respectively [17]. Removal efficiency by filtration treatment was affected by multiple mechanisms, such as straining, interception, gravitational sedimentation, diffusion, and particle attachment/detachment.

In addition to the traditional methods (filtration, coagulation, centrifugation, flocculation, and disinfection) used to remove MPs/NPs from drinking water, new effective methods involve the use of microorganism-based degradation, membrane separation with a reactor, and photocatalysis [107].

A membrane bioreactor can be used as an effective technology in the treatment of drinking water. For instance, the removal of PVC with a concentration of 10 particles/L daily and size < 5 μm from synthetic water by using a membrane bioreactor was studied by Li et al. [91]. Besides a removal rate of organic matter and ammonia over 80% and 95%, respectively, higher membrane fouling and irreversible membrane fouling were observed in the case of PVC contamination.

An eco-friendly and sustainable method for removing MPs from water involves using visible light to activate a photocatalytic process [108]. This approach employs glass fiber substrates to capture low-density MPs, such as PP, while also supporting the photocatalyst material. The process uses zinc oxide nanorods immobilized onto the glass fiber substrates in a flow-through system to break down spherical PP MPs suspended in water via visible light irradiation. After two weeks of this treatment, the average particle volume decreased by 65%. Gas chromatography–mass spectrometry was used to identify the primary by-products of photodegradation, which were found to be mostly non-toxic according to the existing literature.

A new approach that could help remove MPs/NPs from contaminated water involves using algal cells as bio-scavengers [109]. These cells bind the particles to their surfaces or incorporate them into their own cells, filtering them from the water. The polluted biomass can then be further processed downstream through microalgal cultivation, together with sustainable biofuel production, ultimately destroying the MPs/NPs.

All the methods used to remove MPs/NPs from DWTPs require special attention, considering the emergence of new MPs/NPs during the distribution network to tap water consumers. Also, it is important to note that these technologies may not remove all MPs from drinking water sources, and a combination of treatment methods may be necessary to achieve the desired level of removal.

### 3.2. Analytical Methods for Monitoring Micro- and Nanoplastics in Drinking Water Sources

A critical evaluation of applicable methods used for the identification and quantification of MPs/NPs was recently performed by Cella et al. [110], Ivleva et al. [111], Liu et al. [112] and Lee et al. [113]. Criteria used for the quantitative evaluation of the quality of MP concentration data were well reviewed by Koelmans et al. [114]. In this section, the sensitive methods based on mass or particle size needed for identification and quantification of MPs from DWTPs, tap water and bottled drinking water sources have been reviewed.

Table 1 shows the characteristics of MPs/NPs found in drinking water sources in terms of size, concentration, polymer type or morphology monitored by different analytical techniques.

#### 3.2.1. Methods Based on Mass Determination

The most used laboratory methods for MP/NP identification based on mass determination are Pyr-GC/MS [85,121,128,129], TD-GS/MS [122], SP-ICP-MS [9], surface-enhanced Raman spectroscopy (SERS) [130], ICP-MS [131], MALDI-ToF/MS [132], TD-PTR/MS [133], quantitative proton nuclear magnetic resonance (^q1^H NMR) HPLC [134], and differential scanning calorimetry (DSC) [135]. Scarce results have been reported regarding the detection of MPs in drinking water sources, possible due to a lack of standardized methods, limits of quantification, high cost of analytical tools, as well as variety of plastic pollutants in drinking water sources [76]. The analytical methods developed for the investigation of NPs are still uncertain, mainly due to limitations in laboratory workflow and the low sensitivity of analytical tools, which result in limited signals [136,137].

Funck et al. [128] developed a method for quantification of MPs from 3500 L water collected from DWTP by Pyr-GC/MS analysis using cascade filtration with mesh sizes of 100 μm, 50 μm and 10 μm and a platinum filament having dimensions of 20 mm × 5 mm for sample application. Thus, the quantification limits for PS and PE were 0.03 μg and 1 μg with a relative standard deviation of 11%.

A recent study has reported the quantification of NPs found in bottled water down to 1 nm [122]. Huang et al. [122], developed a new method based on thermal desorption (TD) into a gas chromatography–mass spectrometry (GC/MS) system coupled with tangential flow ultrafiltration (TFU) and evaporation techniques. The principle consisted in the concentration of NPs in retentate fluids by dewatering and desalting, thus preventing the loss of solids. The authors demonstrated the presence of PET-degradation products (from bottles) during a thermal mechanism and exposure to light. The advantages of this TD-GC/MS technique compared with other techniques were a higher resolution analysis without the use of organic solvent, low sample volume for NP determination, and analysis time (27 min per sample) [122].

Li et al. [121] successfully used a sequential filtration with inorganic filters, followed by identification of chemical groups using a micro-zone through atomic force microscopy (AFM) coupled with infrared spectroscopy (AFM-IR), and, finally, employed Pyr-GC/MS for the identification of polymer in tap water [121]. The authors quantified NPs ranging from 58 nm to 255 nm as polyolefins, PS, PVC, PA, and some plastic additives.

A suspension of PS22 model NPs conjugated with AuNPs@gel at a concentration of 1 × 10^12^ particles/L was used for the development of the SP-ICP-MS method to quantify NPs up to 1 µm and a concentration of 8.4 × 10^5^ NPs/L in drinking water and tap water samples [9]. The strategy of using SP-ICP-MS for the detection and quantification of NPs in water sources is based on the oxidation signature of aged plastic debris, and the conjugation of carboxyl groups from the surface of NPs particles with functionalized positively charged metal (gold)-containing NPs (AuNPs).

**Figure 3:**
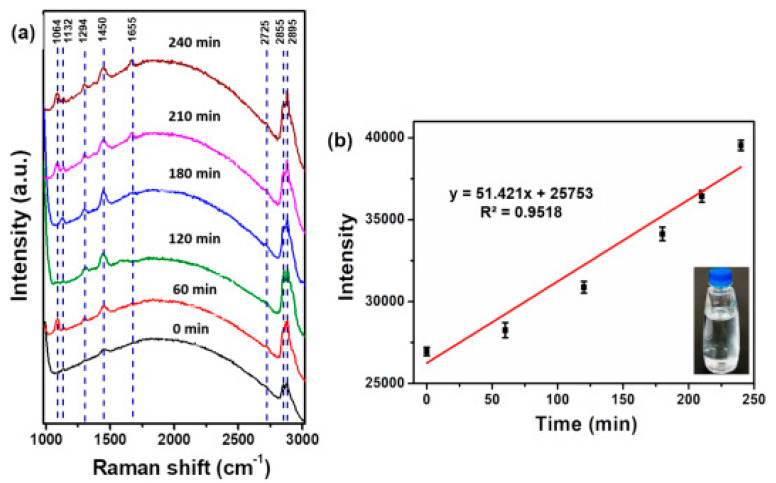
(**a**) SERS spectra of PE particles released from bottled mineral water after four irradiation times: 0, 60, 120, 180, 210 and 240 min. (**b**) Linear regression of the peak absorption band at 2895 cm^−1^ versus time over the full 240 min experiment. Each spectrum was determined three times. Reprinted with permission from [130].

Lin et al. [130] confirmed that PE particles were gradually released over time in plastic cup and bottled mineral water samples during irradiation by using of SERS. Analysis of the signal spectrum collected in 15 s revealed that the PE concentrations measured for plastic cups and bottles were 3751 ± 0.19 ng/mL and 1522 ± 0.21 ng/mL, respectively, after 240 min. The detection limit for NPs was 1.6 ng/mL when copper oxide/silver NPs (CuO/Ag NPs) were used as SERS substrate (Figure 3). Similar concentrations of MPs ranging from 1.67–2.08 µg/L were found in tap water by using Pyr-GC/MS [121].

A nanowell-enhanced Raman spectroscopy (NWERS) substrate, composed of self-assembled SiO_2_ sputtered with silver films (SiO_2_ PC@Ag), was developed for PS NP detection from tap and bottled drinking water, with a size < 200 nm and a limit of detection (LOD) of 5 µg/L [138].

Realistic PET NPs from bottled water labeled with an iridium-containing organic molecular agent were detected in liver, spleen, lung and kidney via inductively coupled plasma mass spectroscopy (ICP-MS) [131].

Asymmetric flow-field flow fractionation (AF4) and multiangle and dynamic light scattering (DLS-MADLS) methodologies were used by Villacorta et al. [139] for monitoring PET NPs from plastic water bottles. To tackle the real NP sample without metal contamination, the authors used diamond burrs to obtain uniform and representative samples with a size of about 100 nm for the investigation of potential health risks.

#### 3.2.2. Methods Based on Particle Determination

The best-known methods used in the laboratory for MP/NP identification based on particle determination are FTIR [16,119,121,124], µ-FTIR [62,115], µ-Raman [116,123,125], SERS [130], SEM [65] and DLS [88]. An LDIR chemical imaging system is also a useful analytical tool based on the μ-FTIR technique for detection of the number of MPs, polymer types and sizes of MPs in the case of diameters > 20 μm [118,126].

An interesting review on the statistics of MP presence in drinking water sources, published in 2020, reported an average concentration of MPs in conventional water sources of 2.2 × 10^3^ items/m^3^, with an identified particle size usually > 50 µm [86].

Pivokonsky et al. [16] studied the removal capacity of plastic particles at three DWTPs and their concentrations in treated water. Plastic fragments and fibers were the most common shapes found in drinking water. These fragments are supposed to occur in drinking water during the breakdown of macroplastics, while fibers could be supplied from the discharge of washing machines into sewage waters.

The aggregation of positively charged PS NPs with a diameter of 15 nm and a specific surface area of 40–60 m^2^/g at a concentration of 10 mg/L introduced into three types of commercial bottled mineral waters was observed at a point of zero charge (pzc), pH_pzc_ of 9.9 ± 0.1 [88]. Positively charged PS NPs have been reported to be difficult to coagulate and remove from surface and mineral drinking waters [88].

Various treatments applied in DWTPs, as well as different analytical tools and geographic location, led to different concentrations of MPs. µ-FTIR spectroscopy is a suitable technique for the identification and classification of MPs, with sizes ranging between 25 and 500 µm [62]. Microplastics, such as PE, followed by PS, and PET, were identified using µ-FTIR in 17 out of 30 samples [62]. In treatment sludge from a DWTP (Germany), a volume of 1000 m^3^ raw water intake was analyzed by µ-Raman spectroscopy to detect the abundance and type of MPs with sizes > 50 μm over a period of 3 h. A figure of 196 ± 42 MPs/m^3^ was found [116]. Similar concentrations (338–628 MPs/L) were reported from another three DWTPs by using FTIR and Raman spectroscopy [16]. µ-Raman and SEM allowed the detection of 6614 ± 1132 particles/L, from which the predominant types were PET, PE, PP, polyacrylamide (PAM), PS and PVC [68]. Interesting was the presence of PAM MPs in the treated water, denoting its use in the removal of suspended particles. However, it is considered that the product derived from PAM is more toxic than to polymers and the dosage of PAM should be according to the regulations in force [83]. Small concentrations of MPs were detected by using LDIR and optical microscopy techniques at various stages of a DWTP (2 MPs/L) [118], purification and density separation followed by FTIR and SEM revealing concentrations of 0.022–0.051 MPs/L [119]. Additionally, µ-FTIR coupled with an FPA detector detected 0.7 MPs/m^3^ [115].

The concentration of MPs in water can vary depending on the source and treatment processes. Tong et al. [117] conducted a study in which particles larger than 300 µm were found in 38 tap water samples. Fragments were found to be the most common morphotype in most tap water samples, followed by fibers and spheres. Plastic particles found in tap water may originate from the use of PE and PP in pipes used for distributing drinking water. It is possible that the water treatment process does not effectively remove or break down larger particles. A comparative study addressing the amount of MPs detected in DWTPs and tap waters showed high concentrations of MPs in tap waters compared to the levels found in DWTPs [119], implying the possible release of MPs from plastic pipes.

According to the research conducted by Zhang et al. [17], 81% of MPs were found in 159 samples of tap water, while 93% of MPs were detected in 259 samples of bottled drinking water from 11 different brands. MPs/NPs can contaminate bottled drinking water when the screw cap is opened and closed [101,123] or due to degradation compounds associated with PET [122]. The larger plastic particles found in bottled drinking water, compared to those found in tap water, are attributed to the types of material used for the bottles and caps [16]. For example, the opening of a plastic bottle can produce 0.46–250 MP particles/cm [140]. Weisser et al. [124] also found that 81% of MPs detected in bottled drinking water were attributed to abrasion of the PE-based cap sealing material. Thick-necked plastic bottles were found to release more MPs than thin-necked glass bottles [126]. Plastic food packaging is also a considerable source of the release of MPs/NPs [22,50,110,141]. On average, isolated MPs weighing 3 mg to 38 mg were detected in each consumer plastic food container [141].

The dimension of plastic particles detected in bottled drinking water varies from 58 to 255 nm [121], 66–605 nm [122], and ≥11 µm [124]. In a remarkable study conducted by Oßmann et al. [125], plastic particles < 5 µm in size were identified in water packaged in single-use PET, reusable PET and glass bottles. The MPs found in glass bottles were explained based on the age of the bottle. The authors prepared the water samples for µ-Raman spectroscopy analysis by mixing an equimolar amount of 250 g/L ethylenediaminetetraacetic acid tetrasodium salt (EDTA) corresponding to Ca^2+^ and Mg^2+^ ions in water with the water sample, for 15 min, by adding 3 mL of 100 g/L sodium dodecyl sulfate (SDS), then vacuum filtration through an aluminum-coated PC membrane filter with pore size of 0.4 µm. Of interest is the high concentration of 384 ± 468 MPs/L found in the blank sample, in which PP, PS, PE and PET were identified.

**Figure 4:**
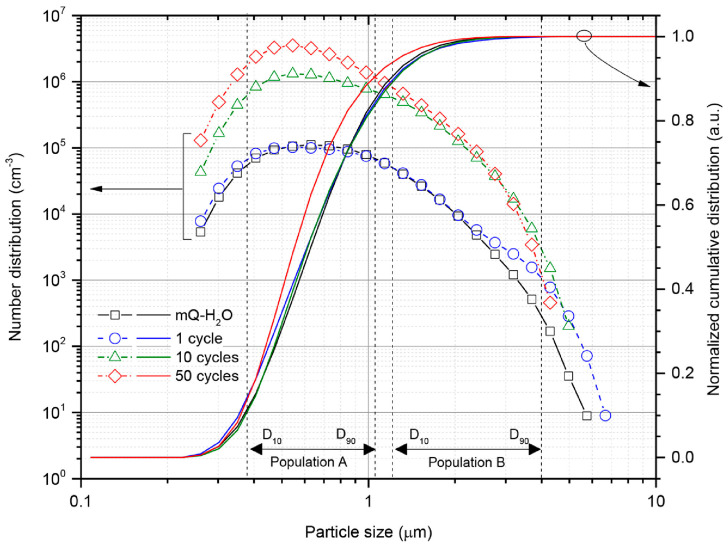
SPES histograms performed for measuring the size of NPs released from three opening–closing cycles—1, 10, and 50 times—at a volume of 15 ± 0.3 mL per sample. Dotted lines are the boundaries of population A and B particle size distributions indicated in terms *D_10_* and *D_90_*as calculated from SPES histograms [123].

Single-particle extinction and scattering (SPES) determines the number and size distribution of particles. Quantitative and qualitative analyses of NPs released in drinking water plastic bottles under realistic conditions show that the PE sealing of the bottles released particles with a size distribution ranging from several hundreds of nanometers to about 1 µm, and estimated a mass release in the order of a few tenths of nanograms per opening/closing cycle [123]. The physicochemical characteristics of the produced secondary NPs were influenced by mechanical stress, making their identification difficult. The combination of SPES and µ-Raman represents the minimum set of techniques required for the application of NP quantification and identification methodologies in simple matrices, such as drinking water. The size distribution of NPs released from a package measured using the SPES technique showed 10–90% distribution for population A of *D_10__A* = 0.38 ± 0.03 µm and *D_90__A* = 1.04 ± 0.14 µm, respectively [123] (Figure 4).

A novel qualitative characterization of PET MPs derived from PET bottled water was proposed by Asamoah et al. [142], who analyzed the optical surface roughness together with the speckle contrast of the rough MPs. The use of an optical sensor prototype to detect the flat, nearly flat, curved, and rough MPs prepared from commercial PET plastic and PET bottles in water is promising for the development of a portable optical sensor capable of real-time detection of MPs and NPs in an aqueous environment. The optical behavior evaluated by specular reflection technique detected the residence time of MPs in water, the rate of pollutant adsorption, as well as the hydrodynamics and aerodynamics of MPs.

Overall, these methods based on particle determination provide valuable information for identifying and quantifying MPs and NPs in drinking water. However, it is important to use multiple analytical techniques and to validate the results with other methods to ensure accurate and reliable identification.

### 3.3. Potential Toxicological Effects of Micro- and Nanoplastics from Drinking Water Sources on Human Health

Assessing the clear health effects of MPs/NPs on marine organisms and humans is a huge challenge among researchers [64,79]. The potential human health risks posed by exposure to MPs and NPs are continuously explored, but few published papers have been related to direct human health effects. Most research reports have involved animal studies, mathematical modeling or in vitro cell culture, so there is still a lack of data on direct human exposure and effects.

Since the existence of MPs and NPs has been proven in food, water, air and consumer products, human exposure to MPs/NPs can occur through ingestion (primary route), inhalation and dermal contact [143]. MPs/NPs could be initially ingested by marine species (lower trophic levels) and further bioaccumulate, potentially leading to human exposure. The existence of MPs and NPs in organisms can cause oxidative stress, cytotoxicity, neurotoxicity, inflammatory lesions, increased uptake or translocation, metabolic disturbances, reproductive issues and increased cancer risk in humans [144]. Health risks depend on the concentration, exposure period, route of exposure and the physicochemical properties of the particles [66].

The entrance of MPs and NPs into the human body could additionally involve the introduction of other associated substances incorporated into plastics, such as plasticizers, stabilizers, opacifiers, flame retardants, antistatic, conductive or medical additives or substances considered endocrine disruptors [145], which can migrate from the matrix due to depolymerization and leaching processes, leading to possible cytotoxicity and inflammatory response. A study performed by Tisler and Christensen [146] on reusable plastic (PE and biodegradable PE) and glass sport bottles revealed the migration of >400 plastic-related compounds over 24 h into drinking water, among which plasticizers, antioxidants, and photoinitiators were predominant. Among the toxic chemical additives potentially existing in MPs/NPs that impose high concern for human health are phthalates, bisphenol A (BPA), brominated flame retardants (BFR), triclosan, bisphenone and organotins [147]. BPA is an important chemical used as a monomer for PC [148], antioxidant or plasticizer in PP, PE, PVC, epoxy resins and coating used to line the packaging of food and beverage cans [149,150]. It has been reported that BPA migrates out of PC, epoxy resins and other consumer plastics [151] and may contaminate food products and drinks [152,153], producing adverse effects on human health, such as liver and pancreatic function alternation and respective changes in insulin resistance. BPA leaching also affects the development of offspring in the wombs of pregnant women, causing issues with brain function [154,155] and inhibiting thyroid hormone-mediated transcription by acting as an antagonist [156]. Some authors reported the onset of obesity and cardiovascular disease [157,158].

In respect to the simultaneous assessment of three surfactants, 4-nonylphenol (4-NP), BPA, and triclosan (TCS) in bottled water, the data show that an adult can ingest 340 ng/day of 4-nonylphenol (4-NP), 165 ng/day of BPA and 7 ng/day of TCS [148]. While 4-NP can come from HDPE and PVC containers, BPA can come from PC baby bottles and TCS is widely used as a preservative and antimicrobial agent in personal care products [159]. An adult could ingest 1410 ng/day of 4-NP, 148 ng/day of BPA, and 10 ng/day of TCS when drinking tap water [148]. Daily BPA intake for infants was three times higher than that for adults [148]. According to Directive 2011/8/EU, the import of PC bottles from countries outside of the European Union was prohibited from 1 June 2011, and a total ban on the use of BPA for manufacturing baby bottles was introduced in the European Union on 1 March 2011 [151].

While BPA is not intentionally added during the manufacturing of PET bottles, studies have suggested that the bottle cap, recycled PET or exposure to heat and ultraviolet radiation could lead to its release in water [160]. Another study reported the presence of BPA in PET bottled drinking water, and leaching increased with temperature [151]. For PC bottles exposed to hot water, an increase in the rate of BPA migration up to 55-fold was observed [155]. While there is not a significant risk to human health from exposure to BPA in drinking water, attention should be paid to the cumulative daily dose in the body.

Phthalate esters are employed as plasticizers to improve the flexibility of various plastic materials, much used in manufacturing PVC and plastisol [161] and PET bottles [162]. The potential harmful effects of phthalate esters on human health consist in abnormal sexual development and birth defects [163], while they have also been reported to induce adverse cellular changes in fish [147]. The presence of 17 phthalate esters was reported in drinking water stored in PET bottles without any threat to human health [162]. In other study [164], the phthalate ester identification was assigned to cross-contamination from the laboratory. However, it is recommended that an investigation into the additives released from water plastic bottles from a toxicological perspective should be carried out over the long term.

According to the results of in vitro tests on marine species, MPs have been shown to accumulate in the gills, stomach, and metabolic organs of crabs [165].

MPs larger than 10 μm are unlikely to be transported through an intact intestinal barrier [7,166]. In this regard, Gao et al. [131] suggested that PET NPs produced by mechanical action on bottled water could not be identified in the liver, spleen, lungs, or kidneys of mice, which indicates that they cannot penetrate the intestinal barriers when injected intravenously.

Animal model studies reported the accumulation of NPs in the placenta [167]. Aghaei et al. [74] were the first authors to demonstrate that the MPs and NPs in drinking water pose risks to human pregnancies in late gestation. After exposing fetuses to PS both as MPs and NPs with sizes of 5 μm and 50 nm, respectively, in a concentration of 10^6^ ng/L, a 12% decrease in fetal weight was observed. However, the risks to embryo–fetal development associated with unintended ingestion of MPs/NPs require extended investigations.

Once they enter the body, MPs might translocate to distant tissues through the circulatory system, causing a systemic inflammatory response, blood cell cytotoxicity through internalization [168], pulmonary hypertension [169], vascular inflammation or occlusions [170], and decreased organ function, as well as an increased risk of neoplasm due to deoxyribonucleic acid (DNA) damage [171].

In another paper, the ability to induce reactive oxygen species (ROS) and DNA damage were evaluated after the exposure of two human lymphoblastic cell lines to PET NPs removed from drinking water bottles [139]. Preliminary research revealed cellular uptake, but without the induction of significant biological effects, and thus no potential health hazard.

On the contrary, Ji et al. [172] demonstrated that the size of PET NPs (20 nm, 60–80 nm, and 800 nm) obtained by a process of mechanical breakdown and dispersing agents (SDS and bovine serum albumin (BSA)) used for NP stabilization has an important role in in vivo toxicity studies. Thus, PET pieces cut from mineral water bottles have been shown to affect the hatching rate, heart rate, and ROS generation in the development of zebra fish. The BSA-dispersing agent was found to induce a higher level in heart rate abnormalities and more severe oxidative damage from the PET NPs than SDS.

**Figure 5:**
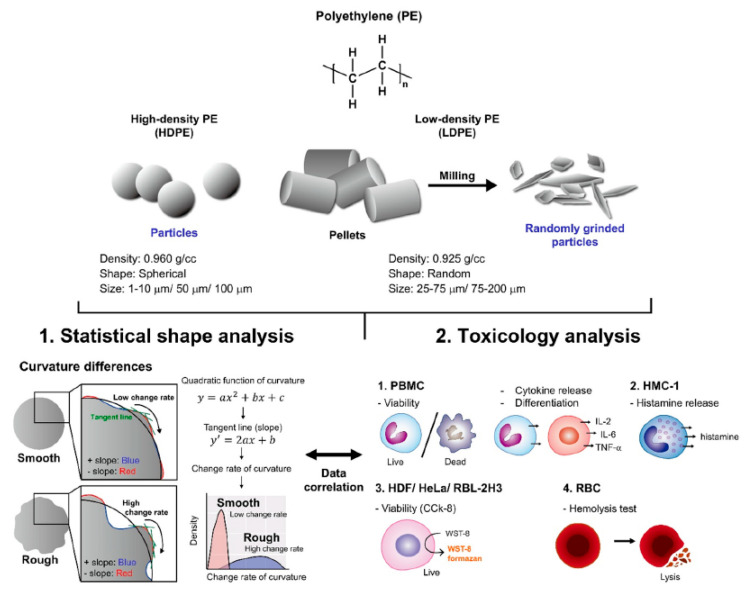
Correlation of physical aspects of PE MPs with statistical data and in vitro toxicity results. CCK-8: cell counting kit-8; WST-8: water-soluble tetrazolium salt-8. Reprinted with permission from [175].

Cell damage caused by extrinsic toxic plastic materials can be evaluated by some typical examinations, such as rupture of the cellular membrane by strong positive charge [173], interference with DNA synthesis, or organelle activities after uptake [174], resulting in cell death due to necrosis or apoptosis [175]. Choi et al. [175] examined the in vitro toxicity of PE MPs of different shapes and sizes, such as 1–100 μm for HDPE and ranging between 25–75 μm and 75–200 μm for LDPE, on cultured cells, including immune cells (human mast cells [HMC-1], peripheral blood mononuclear cells [PBMCs], red blood cells [RBCs], rat basophilic leukemia cells [RBL-2H3]), non-immune cells (cervical cancer cells [HeLa]), and human dermal fibroblasts [HDFs]. Their results showed that the HDPE particles with relatively smooth surfaces did not produce significant cytotoxicity in cells, but induced an immune response in PBMCs and enhanced PBMC differentiation, while the LDPE particles with sharp edges (higher curvature change) caused increased cytotoxicity under direct cell–microplastic interaction, inflammatory response, hemolysis, and ROS production at high concentrations (Figure 5).

Magri et al. [176] mentioned that combining of alternative methodologies, such as metabolomics, with standard biological assays (i.e., cell viability and ROS production) is an important approach to acquire preliminary valuable information on cellular metabolism to facilitate the prediction of potential effects of plastic NPs on human health. Metabolomics is a powerful tool for studying cellular metabolism and can be used to identify changes in metabolite levels in response to exposure to plastic NPs. The authors used pulsed-laser ablation of solid PET films in water to form PET NPs (size distribution in the range of 10 and 80 nm) of similar surface and shape irregularity, broad size distribution, and chemistry to those of particles in the environment [104]. They found a binding capacity of about 3% *w*/*w* NPs for PET NPs with levofloxacin, an antibiotic defined as an emerging contaminant in aqueous environments and demonstrated that these nanoclusters are not toxic to heterogeneous human epithelial colorectal adenocarcinoma cells (Caco-2) in the short term, but affect the cells metabolism as a compensatory response to oxidative stress, suggesting long-term risks.

The cytotoxicity of PE and PS MPs was evaluated in vitro in cell lines T98G and HeLa (cerebral and epithelial human cells) exposed for 24–48 h to different contaminant levels of 10 ng/mL to 10 μg/mL under the same conditions [177]. Oxidative stress explains the toxicity of PE and PS MPs at the cell level, being significant in the case of PE MPs for T98G; however, PS showed higher ROS generation in both cell lines.

The effect of engineered aminated PS NPs (model particles) on human liver HepG2 hepatocytes was investigated by Banerjee et al. [178]. They found that the uptake of NPs with sizes of 50 or 100 nm by HepG2 liver cells was higher than 1000 nm particles. Additionally, short-term exposure to aminated PS NPs resulted in cell toxicity, while those with sizes between 500–5000 nm induced apoptosis [178].

Organic matter found in commercial drinking water was analyzed by various analytical methods, such as solid residues, after freeze-drying treatment of drinking water, using analytical methods including dry ash, centrifugation, Raman analysis, and electron microscopy [179]. The study found a concentration of 0.25–2.0 mg/L organic matter. It was proved that the stress applied to the plastic bottle did not significantly increase the concentration of organic matter within one month, and the organic matter did not affect the viability of human intestinal cells [179]. However, the study suggested that prolonged exposure to nanoscale material in drinking water could potentially lead to the accumulation of NOM in the human body over time.

The potential toxicological effects on test organisms, as well as the use of biodegradable MPs as vector for other chemical pollutants and microorganisms, have been reported [43]. However, there is a serious concern regarding the management of bioplastics and MP contamination, particularly with regard to fragmentation, degradability, and toxicity, and their interaction with other chemical contaminants present in aqueous media [41]. Since most MPs/NPs are hardly biodegradable or non-biodegradable, they clearly remain intact inside the living organisms for a long time. The prolonged exposure of the human or other organisms to MPs/NPs may lead to chronic irritation, inflammation, cellular proliferation, and necrosis, and may compromise immune cells [144,180].

Comprehensive studies are needed to establish a clear health-risk assessment for exposure to MPs and NPs, as it is fundamental to set the reference method of investigation to monitor each source of human intake.

## 4. Conclusions and Future Perspective

The removal efficiency of MPs/NPs from drinking water treatment plants varies depending on the treatment processes applied. The size of MPs detected in DWTPs ranges from 1.2 µm to 2000 µm. Most studies on MPs/NPs have focused on investigating PS NPs models due to their prevalence as debris in water. However, further analytical development is required to monitor real NPs that have been isolated and/or preconcentrated from more complex matrices. Few studies have reported the detection of NPs present in drinking water bottles. A standard that is kept under the same conditions as drinking water is needed to quantify the concentration of NPs in drinking water. Additionally, studies should also investigate the interaction between NPs and other sources, such as food.

In the future, the amount of MPs/NPs in tap water and bottled drinking water are estimated to unfortunately expand as a consequence of the continued degradation and fragmentation of plastics in the environment. The manufacturing technology of reusable drinking bottles (PET, glass) should be carefully examined to decrease the concentration of MPs/NPs in the water packaged in the respective containers. The European Chemicals Agency (ECHA) has proposed far-reaching restrictions on the use of MPs in products marketed in the EU to reduce their release into the environment, as a part of the circular economy plan [35]. The risks associated with MP/NP pollution derived from drinking water sources should be mitigated by applying legal, technical, and social measures [6,7,8,30,63,73,132], such as:

- setting sampling and monitoring standards for MPs/NPs
- reducing the production of non-biodegradable plastic items
- reducing single-use plastic
- implementing the circular economy using biodegradable plastic items
- using the “refuse, reduce, reuse, and recycle” concept
- innovation for plastics that do not need reusable, recyclable, or compostable materials
- total removal of MPs/NPs from DWTPs
- designing innovative packaging technologies to unscrew bottle caps in other ways, such as easy-to-open caps
- the use of bio-inspired technology related to biomimetics involving the design of advanced systems or devices inspired by nature, where principles from interdisciplinary fields such as engineering, chemistry and biology are applied to the development of materials, synthetic systems or instruments with functions that mimic biological processes [181]
- raising people’s awareness of the toxicological effects of MPs/NPs.

In addition to improving multiple interventions and management to prevent the release of plastic related to the removal of MPs/NPs from water infrastructure, other effective measures are necessary to eliminate MPs/NPs directly at the source. A green prevention technology was proposed by the GoJelly Project, which developed a prototype microplastics filter for commercial and public use, employing jellyfish mucus as the main raw material [182]. The use of special household water systems, such as Lint LUV-R and Showerloop, is effective in filtering out microfibers at the domestic level [182]. Synthetic fibers could also be captured in the washing machine by means of laundry balls, such as Cora Ball and Fibre Free [183].

Research is still needed to establish an acceptable upper limit for the concentration of various NPs in drinking water through toxicity assessment, unification of various analytical protocols for NP/MP identification and development of testing standards. In the meantime, it should not be assumed that any level is safe. Toxicity assessment of various MP and NP types of different chemical composition, size and shape should be studied promptly to evaluate concerns regarding human exposure to these particles. Extensive studies are required to establish an explicit health-risk assessment for exposure to MPs and NPs, as a clear distinction of plastic particles entering the human body from different sources (water, air, food, drugs, skin) cannot be achieved. Awareness of MP/NP pollution of drinking water sources should increase among European regulatory bodies, decision-makers, practitioners and researchers.

## Figures and Tables

**Table 1:** Characteristics of MPs/NPs identified in drinking water sources.

Source	Method	Characteristics of MPs/NPs	Polymer Type	Ref.
Three DWTPs	FTIR and Raman	Concentrations of 443 ± 10, 338 ± 76 and 628 ± 28 particles/L	PET, PP, PE	[16]
1200 L to 2500 L water from DWTPs (sampled in 2014 in Germany)	µ-FTIR microscopy coupled to a FPA detector	Four blank samples contained 45 ± 22 fibers, 18% were black and 78% were transparent; DWTPs show 0.7 fibers/m^3^, with sizes ranging from 50 to 150 μm	Control: PP, SAN DWTPs: PE, PA, PES, PVC or epoxy resin	[115]
DWTP, Germany	µ-Raman	MPs size varying from 50 to 5000 μm; Shape of MPs: 83.3 % fragments and 16.7% fibers	37.8% PE, 31% PP and 24.4% PS were the most commonly found plastic in the analyzed samples	[116]
38 samples were collected from different tap waters from China	µ-Raman	Concentration of MPs was varying from 0 to 1 247 particles/L Distribution of MPs according to different size classes was: 31.25 to 100% for 1–50 µm; 1.47 to 31.25% for 50–100 µm; 1.72 to 31.25% for 100–300 µm; 1.18 to 7.69% for 300–500 µm; 1.72 to 11.76% for 500–5000 µm	26.8% PE, 24.4% PP, 22% compounds of PE and PP, 7.3% polyphenylene sulfite (PPS), 6.5% PS, 3.3% PET, and 9.8% other	[117]
DWTPs	LDIR, optical microscopy	MPs decreased after pre-treatment (80–99%); Size of MPs ranging from 20 to 500 µm; concentration of 2 MPs/L	PA, PET, PE, rubbers, chlorinated PE	[118]
Two DWTPs and ten tap water samples (from Iran)	Density separation techniques, digestion, observation, µ-Raman and FTIR, and SEM	An average of 22–51.8 MPs/m^3^ for DWTPs; A high concentration of particles in tap water (85–390 MPs/m^3^) compared to those found in DWTPs	PS	[119]
Two tap water samples (collected from Saudi Arabia)	µ-FTIR	First sample: 1.8 MPs/L Second sample: <LOQ	PE	[62]
159 samples of tap water collected between January and April of 2017, from 14 countries	FTIR	Concentration ranging from 0 to 61 MPs/L, with an overall mean of 5.45 MPs/L; 98.3% MPs were identified as fibers, and the remaining particles were identified as fragments or films	Not mentioned	[120]
Tap water sample (from China)	FTIR, AFM-IR and Pyr-GC/MS	The most frequently occurring particles had a size ranging from 58 to 255 nm, and a concentration between 1.67–2.08 µg/L	PE, PP, PS, PVC, PA	[121]
Two brands of bottled water in PET bottles	TD-GC/MS combined with TFU; super-resolution optical nanoscopy with microsphere lens; DLS	Size ranging between 66–605 nm	Degradation products of PET: phthalate derivatives and ethyl *p*-ethoxybenzoate	[122]
Mineral water bottles (0.5 L) consisted of transparent PET, with cap made of white HDPE	SPES and µ-Raman	*Size distribution: fewer than 10% of particles have a dimension of* 0.38 ± 0.03 µm, and *fewer than 9% of particles have a dimension of* 1.04 ± 0.14 µm	HDPE, PET	[123]
Four mineral water bottles	FTIR	Concentrations ranging from <1 MP/L to 317 ± 257 MPs/L, with particle sizes ≥ 11 µm	PE, PP, PS, polyester, PVC, EvOH, and PA	[124]
32 samples were collected from 21 different brands of mineral waters (from Bavarian location)	µ-Raman	Single use PET bottles: 2649 ± 2857 MPs/L Reusable PET bottles: 4889 ± 5432 MPs/L Glass bottles: 6292 ± 10,521 MPs/L Single and reusable PET: 95% of the plastic particles < 5 µm and 50% < 1.5 µm Glass bottle: ~15% of plastic particles were between 5 µm and 10 µm, and ~7% > 10 µm	PET for PET water bottle; PE (46%), PP (23%) and a styrene-butadiene-copolymer (14%) for glass water bottle	[125]
Ten mineral waters, either still or sparkling, in PET plastic bottles (from Catania, Italy)	SEM, density, statistical analysis	MPs with a mean diameter of 2.44 µm ± 0.66 µm were detected on PET surface	Not mentioned	[65]
Drinking water stored in PC and PP bottles (from China)	LDIR chemical imaging system, TEM	53 to 393 particles/mL during 100 opening/closing cycles	PC, PP	[126]
63 drinking water samples collected from decentralized refill kiosks in the Mexico City	ATR-FTIR	11 to 860 MPs/L from which: 65% were fibers, 28% fragments, and 7% films	PET, PA, vinyl polymers, polyacetals, cellophane	[127]

Focal plane array (FPA), styrene acrylonitrile (SAN), tangential flow ultrafiltration (TFU), dynamic light scattering (DLS), single particle extinction and scattering (SPES), atomic force microscopy–infrared spectroscopy (AFM-IR), ethylene vinyl alcohol (EvOH), polyamide (PA), polycarbonate (PC), laser direct infrared (LDIR), ATR-FTIR (attenuated total reflectance–Fourier-transform infrared spectroscopy).

## Data Availability

The data presented in this study are available on request from the corresponding author.

## Supplementary Materials

The following supporting information can be downloaded at https://www.mdpi.com/article/10.3390/polym15112425/s1. Table S1: Physicochemical methods used to remove MPs/NPs from drinking water.

## List of Acronyms and Abbreviations

MPs	microplastics
NPs	nanoplastics
DWTPs	drinking water treatment plants
Pyr-GC/MS	pyrolysis–gas chromatography–mass spectrometry
RAC	Committee for Risk Assessment
SEAC	Committee for Socio-Economic Analysis
EPS	extracellular polymers
PFAS	per- and polyfluoroalkyl substances
WHO	World Health Organization
ATSDR	Agency for Toxic Substances and Disease Registry
PS	polystyrene
PVC	polyvinyl chloride
CFS	coagulation–flocculation–sedimentation
TD-PTR/MS	thermal desorption–proton transfer reaction–mass spectrometry
µ-FTIR	micro-Fourier-transform infrared spectroscopy
µ-Raman	micro-Raman
TD-GC/MS	thermal desorption–gas chromatography–mass spectrometry
SP-ICP-MS	single particle inductively coupled plasma mass spectroscopy
SERS	surface-enhanced Raman spectroscopy
ICP-MS	inductively coupled plasma mass spectroscopy
MALDI-ToF/MS	matrix-assisted laser desorption/ionization time-of-flight mass spectrometry
DLS	dynamic light scattering
SEM-EDX	scanning electron microscopy–energy-dispersive X-ray spectroscopy
TEM	transmission electron microscopy
PET	polyethylene terephthalate
NOM	natural organic matter
polyDADMAC	diallyldimethylammonium chloride
PE	polyethylene
PACl	polyaluminum chloride
Al_2_(SO_4_)_3_	aluminum sulfate
AlCl_3_	aluminum chloride
FeCl_3_	iron chloride
NaHCO_3_	sodium bicarbonate
PC	polyamine-coated
ER	elongated-rough
ES	elongated-smooth
SR	spherical-rough
SS	spherical-smooth
GAC	granular activated carbon
Pd	palladium
DOM	dissolved organic matter
IONPs	iron oxide nanoparticles
LOQ	* limit of quantification *
AF4	asymmetric flow field flow fractionation
DLS-MADLS	multiangle and dynamic light scattering
PA	polyamide
PU	polyurethane
HDPE	high-density polyethylene
POU	three point-of-use
IX	ion exchange
MF	microfiltration
PVC	polyvinyl chloride
LDPE	low-density polyethylene
DAF	dissolved air flotation
SAN	styrene acrylonitrile
PES	polyester
PPS	polyphenylene sulfite
FPA	focal plane array
TFU	tangential flow ultrafiltration
LOD	limit of detection
EvOH	ethylene vinyl alcohol
LDIR	laser direct infrared
PC	polycarbonate
AFM-IR	atomic force microscope-infrared spectroscopy
ATR-FTIR	attenuated total reflectance Fourier-transform infrared spectroscopy
NWERS	nanowell-enhanced Raman spectroscopy
ILs	ionic liquids
PAM	polyacrylamide
EDTA	ethylenediaminetetraacetic acid tetrasodium salt
SDS	sodium dodecyl sulfate
BPA	bisphenol A
BFR	brominated flame retardants
4-NP	4-nonylphenol
TCS	triclosan
DNA	deoxyribonucleic acid
ROS	reactive oxygen species
BSA	bovine serum albumin
HMC-1	human mast cells
PBMCs	peripheral blood mononuclear cells
RBCs	red blood cells
RBL-2H3	rat basophilic leukemia cells
HeLa	cervical cancer cells
HDFs	human dermal fibroblasts
Caco-2	human epithelial colorectal adenocarcinoma cells
ECHA	European Chemicals Agency
PAN	poly(acrylonitrile)
PMMA	poly(methyl methacrylate)
PVA	poly(vinyl alcohol)
PEVA	ethylene (vinyl acetate) copolymer
AC	activated carbon
