# Supplementary material for: Insights into Anthropogenic Micro- and Nanoplastic Accumulation in Drinking Water Sources and Their Potential Effects on Human Health
Source: Polymers (Basel). 2023 May 23;15(11):2425. doi: 10.3390/polym15112425 (PMC10255541; doi:10.3390/polym15112425)
Supplement: Supplementary file 1 [file polymers-15-02425-s001.zip › polymers-2254157-supplementary.pdf]

polymers

Review

Insights into Anthropogenic Micro- and Nanoplastics Accumulation in Drinking Water Sources and Their Potential Effects on Human Health

Maria Râpă [1], Raluca Nicoleta Darie-Niță [2*], Ecaterina Matei [1], Andra-Mihaela Predescu [1], Andrei-Constantin Berbecaru [1*], Cristian Predescu [1]

1 Faculty of Materials Science and Engineering, University Politehnica of Bucharest, 313 Splaiul Independentei, 060042 Bucharest, Romania (maria.rapa@upb.ro; ecaterina.matei@upb.ro; andra.predescu@upb.ro; Andrei.berbecaru@upb.ro; cristian.predescu@upb.ro)
2 Physical Chemistry of Polymers Department, Petru Poni Institute of Macromolecular Chemistry, 41A Grigore Ghica Voda Alley, 700487 Iasi, Romania (darier@icmpp.ro)

* Correspondence: darier@icmpp.ro; andrei.berbecaru@upb.ro

Abstract:

Currently, anthropogenic microplastics (MPs) and nanoplastics (NPs) are ubiquitous pollutants found in aquatic, food, soil and air environments. Recently, drinking water for human consumption has been considered a significant pathway for ingestion of such plastic pollutants. Most of the analytical methods developed for detection and identification of MPs have been established for particles with sizes > 10 µm, but new analytical approaches are required to identify NPs below 1 µm. This review aims to evaluate the latest information on the exposure to MPs and NPs through daily human consumption of water, particularly drinking tap water and commercial bottled water, and their potential dermal, inhalation and ingestion effects on human health. Emerging technologies used to remove MPs and/or NPs from drinking water sources and their advantages and limitations were also assessed. The main findings showed that the MPs with sizes > 10 µm were completely removed from drinking water treatment plants (DWTPs); the smallest NP identified by using pyrolysis gas chromatography mass spectrometry (Pyr-GC/MS) has a diameter of 58 nm; contamination with MPs/NPs can occur along the distribution network to tap water consumers, when opening and closing the screw cap or using recycled plastic or glass bottles. To conclude, this comprehensive study highlights the importance of a unified approach to detect MPs and NPs in drinking water, as well as raising the awareness of regulators, policy makers and the public about the impact of these pollutants, which pose a human health risk.

Keywords: drinking water treatment plant; microplastics; nanoplastics; tap water; bottled water; quantification; toxicological effect

Citation: Râpă, M.; Darie-Niță, R.N.; Matei, E.; Predescu, A-M.; Berbecaru, A-C.; Predescu, C. Insights into Anthropogenic Micro- and Nanoplastic Accumulation in Drinking Water Sources and Their Potential Effects on Human Health. *Polymers* **2023**, *15*, 2425. https://doi.org/10.3390/polym15112425

Academic Editor: Graeme Moad

Received: 15 February 2023
Revised: 13 May 2023
Accepted: 19 May 2023
Published: 23 May 2023

Copyright: © 2023 by the authors. Submitted for possible open access publication under the terms and conditions of the Creative Commons Attribution (CC BY) license (https://creativecommons.org/licenses/by/4.0/).

Table S1. Physico-chemical methods used to remove MPs/NPs from drinking water.

Sample	MPs/NPs Characteristics	Method Used	Efficiency of MPs/NPs Removal	Ref.
**Water spiked with fluorescent plastic PE and PS (beads)**	PE: 10–20 μm, 45–53 μm, and 106–125 μm;<br>PS: 180 nm and 1.2 μm	CFS and filtration<br>Alum ($Al_2(SO_4)_3$;<br>PolyDADMAC	CFS: the removal rate increased in the case of polyDADMAC;<br>Filtration: 86.9% ± 4.9% and 99.9% ± 0.1% removal for particles sizes up to 106 μm and in the range of 106–125 μm, respectively	[1]
**Water spiked with PS NPs**	Molecular weight (MW) of 8 × 10^5 Da<br>Concentration of 2.5 μg/L<br>Initial pH of 6.43 ± 0.03	Ozonation and chlorination (sodium hypochlorite, 12.5% Cl_2 by weight)	Achievements after 240 min reaction: 99.9% MW degradation and 42.7% mineralization by ozonation, 7.1% MW degradation and 4.3% mineralization in the case of chlorination	[2]
**NPs (17 mg/L) containing a PAN core labeled with palladium (Pd) and a PS shell in drinking water**	Hydrodynamic diameter (Z-average) of 214.7 nm ± 1.4 nm (polydispersity index: 0.07) and zeta (ζ-potential) of − 41.1 mV ± 0.3 mV (from first synthesis batch)	Ozonation (1.15 mM), sand and activated carbon (AC) filtration	Application of ozonation treatments at concentrations of 0.5, 1 and 5 mg/L, respectively, for 45 min had a minor impact on NPs removal;<br>NPs retention increased with filter length; a sand filter length of 0.1 m and 0.9 m led to NPs removal of 70% and 99.5%, respectively;<br>NPs retention was slow (10%) in the case an AC filter with an initial filter length of 0.9 m	[3]
**Positively charged PS NPs hydrazine (PS NPs (PS-NH-NH_2^+) in water**	Concentration of 4.6 g/L, mean diameter of 124 ± 38 nm (measured by NTA) and 110 ± 25 nm (estimated by SEM)	Filtration<br>Coagulation	GAG filtration: 73.9% ± 2.3% removal efficiency<br>Sand filtration: 54.3% ± 3.1% removal efficiency<br>Overall filtration: 88.1% ± 2.1% removal efficiency<br>The use of PACl resulted in the increase in the removal of NPs to 99.4% ± 1.1%	[4]
**DWTP from Catalonia, Spain**	Outlet, concentration of 0.06 ± 0.04 MPs/L;<br>Inlet, concentration of 0.96 ± 0.46 MPs/L;<br>Particle size of MPs between 20 μm–5000 μm,<br>PS and PES are the main polymers types detected by μ-FTIR	Coagulation and sand filtration	93% ± 5% overall removal efficiency	[5]

**DWTP, India**	MPs were reduced from 17.86 ± 2.66 items/L in the case of raw water to 2.75 ± 0.92 items/ L for treated water (evidenced by ATR FT-IR);<br>Fibers and films/fragments were predominant	Pre-disinfection, coagulation-flocculation, pulse clarification, sand filtration and post-disinfection	85% removal efficiency	[6]
**DWTP, Geneva, Switzerland**	MPs sizes: 63 to 1 000 μm;<br>0 and 4 MPs/m^3 and<br>0 to 3 synthetic fibers/m^3 were found in water;<br>PA, PE, PES, PMMA, PP, PS, PVA and PEVA were the identified MPs	Pre-treatment, sand filtration, activated carbon filtration and pH correction;<br>PACl with a concentration of 0.36 mg Al^{3+}/L was used as coagulant	Coagulant presence led to a higher efficiency of 97% ± 3% and 96% for MPs and synthetic fibers removal, respectively, compared to sand filtration	[7]
**Advanced DWTP located in Yangtze River Delta, China**	MPs >10 um were removed in coagulation/sedimentation<br>PET, PE, PP, PAM, PS, PVC were the identified MPs	Coagulation/flocculation, sedimentation, sand filtration and ozonation integrated with GAC filtration	Overall eficiency of 82.1–88.6%<br>Fibers (51.6–78.9%) and fragments (14.4–38.3%) were the predominant shapes of MPs	[8]

Polyethylene (PE), diallyldimethylammonium chloride (PolyDADMAC), poly(acrylonitrile) (PAN), palladium (Pd), nanoparticles tracking analysis (NTA), scanning electron microscopy (SEM), polyaluminum chloride (PACl), polyester (PES), poly(methyl methacrylate) (PMMA), polypropylene (PP), poly(vinyl alcohol) (PVA), ethylene (vinyl acetate) copolymer (PEVA), polyacrylamide (PAM).

References

1. Zhang, Y.L.; Diehl, A.; Lewandowski, A.; Gopalakrishnan, K.; Baker, T. Removal efficiency of micro- and nanoplastics (180 nm-125 mu m) during drinking water treatment. *Science of the Total Environment* **2020**, *720*, doi:10.1016/j.scitotenv.2020.137383.
2. Li, Y.; Li, J.; Ding, J.; Song, Z.R.; Yang, B.C.; Zhang, C.M.; Guan, B.H. Degradation of nano-sized polystyrene plastics by ozonation or chlorination in drinking water disinfection processes. *Chemical Engineering Journal* **2022**, *427*, doi:10.1016/j.cej.2021.131690.
3. Pulido-Reyes, G.; Magherini, L.; Bianco, C.; Sethi, R.; von Gunten, U.; Kaegi, R.; Mitrano, D.M. Nanoplastics removal during drinking water treatment: Laboratory- and pilot-scale experiments and modeling. *Journal of Hazardous Materials* **2022**, *436*, doi:10.1016/j.jhazmat.2022.129011.
4. Arenas, L.R.; Gentile, S.R.; Zimmermann, S.; Stoll, S. Fate and removal efficiency of polystyrene nanoplastics in a pilot drinking water treatment plant. *Science of the Total Environment* **2022**, *813*, doi:10.1016/j.scitotenv.2021.152623.
5. Dalmau-Soler, J.; Ballesteros-Cano, R.; Boleda, M.R.; Paraira, M.; Ferrer, N.; Lacorte, S. Microplastics from headwaters to tap water: occurrence and removal in a drinking water treatment plant in Barcelona Metropolitan area (Catalonia, NE Spain). *Environmental Science and Pollution Research* **2021**, *28*, 59462-59472, doi:10.1007/s11356-021-13220-1.
6. Sarkar, D.J.; Sarkar, S.D.; Das, B.K.; Praharaj, J.K.; Mahajan, D.K.; Purokait, B.; Mohanty, T.R.; Mohanty, D.; Gogoi, P.; Kumar, V.S.; et al. Microplastics removal efficiency of drinking water treatment plant with pulse clarifier. *Journal of Hazardous Materials* **2021**, *413*, doi:10.1016/j.jhazmat.2021.125347.
7. Velasco, A.N.; Gentile, S.R.; Zimmermann, S.; Stoll, S. Contamination and Removal Efficiency of Microplastics and Synthetic Fibres in a Conventional Drinking Water Treatment Plant. *Frontiers in Water* **2022**, *4*, doi:10.3389/frwa.2022.835451.

8. Wang, Z.; Lin, T.; Chen, W. Occurrence and removal of microplastics in an advanced drinking water treatment plant (ADWTP). *Science of the Total Environment* **2020**, *700*, doi:10.1016/j.scitotenv.2019.134520.
